# Combined Feedback Feedforward Control of a 3-Link Musculoskeletal System Based on the Iterative Training Method

**DOI:** 10.1155/2021/8701869

**Published:** 2021-11-08

**Authors:** Amin Valizadeh, Ali Akbar Akbari

**Affiliations:** Department of Mechanical Engineering, Ferdowsi University of Mashhad, Iran

## Abstract

The investigation and study of the limbs, especially the human arm, have inspired a wide range of humanoid robots, such as movement and muscle redundancy, as a human motor system. One of the main issues related to musculoskeletal systems is the joint redundancy that causes no unique answer for each angle in return for an arm's end effector's arbitrary trajectory. As a result, there are many architectures like the torques applied to the joints. In this study, an iterative learning controller was applied to control the 3-link musculoskeletal system's motion with 6 muscles. In this controller, the robot's task space was assumed as the feedforward of the controller and muscle space as the controller feedback. In both task and muscle spaces, some noises cause the system to be unstable, so a forgetting factor was used to a convergence task space output in the neighborhood of the desired trajectories. The results show that the controller performance has improved gradually by iterating the learning steps, and the error rate has decreased so that the trajectory passed by the end effector has practically matched the desired trajectory after 1000 iterations.

## 1. Introduction

The reaching movement is accounted for a huge part of hand movements. In all these activities, a swift and complex process occurs in the brain, and after processing, the generated control signals are transmitted to body motors, namely, muscles. This complex process in the brain comprises some levels. First, the desired trajectory is determined for reaching an object, and in the second step, the coordinates of the specified trajectory estimated by vision are converted into the body coordinates; in the last step, control commands are sent to the muscular system to go through the desired trajectory. Investigating the body's musculoskeletal system's control mechanism can lead us to develop a robust control technique that can be applied to rehabilitation robotics. The design process and application of the actuators in such robots are similar to the simulation of the human body's neural control system. Many controllers have been introduced and employed to control such systems and produce motions similar to the human movement, which are of different design methods and performances based on their design space (robot task space, joint space, and muscle space) [1, 2]. Each of these spaces has its features and complexity, and as we move from the task space toward the muscle space, it will be difficult to design the controller because of the increasing space order. It should be noted that the controller design in the muscle space should be carried out carefully so that the forces out of the body are ignored, and the model approaches reality. The joint redundancy causes each angle to have no unique solution in return for an arbitrary trajectory of the arm's end effector [3]. Another problem caused by redundancy is the lack of a unique solution for model forces to generate unique torque [4]. Many optimization techniques have been proposed to overcome this problem in classification [5], biology and robotics [6–9].

On the other hand, in everyday life, we can easily carry out the most complex movements with the highest possible accuracy in the presence of the same redundancies with the least possible. One solution to overcome this complexity is that the central nervous system activates a small group of muscles—called muscle synergy [10]—which allows the control of body movements with less computational cost by reducing the required independent degrees of freedom. In recent years, Suetani and Morimoto [11] presented an innovative hypothesis under the title of “Virtual Spring-Damper Hypothesis,” where there is no need to optimize the redundancy criterion to overcome the redundancy problem. However, the previous problem is solved by applying this hypothesis, but due to the redundancy of muscles, we will deal with other problems that require the application of the muscle nonlinear models. In 2013, aiming to find the synergies of reaching and balancing movements on the musculoskeletal system of the hand, Tahara et al. [12] conducted a research study to investigate muscular integrity force data and the data of body receptors (proprioception and vision). The canonical correlation analysis (CCA) method, which follows the natural behavior of the body, was used to obtain the relation between the data related to muscles and body receptors. In a real system, the time delay and noise should be considered in the body's actuator and sensor systems. Hence, this study is aimed at examining the effects of time delay and noise on determining the synergies of the hand's musculoskeletal system. The results of this study not only can be used to understand the biological data of the motor control system but also can be applied as an artificial controller for a high-DOF robot. In this study, the motion equations for the 3-link musculoskeletal system of the human arm and the iterative learning controller are presented in Section 2. The results obtained from the simulation of the ILC (iterative learning control) with the neuro-fuzzy controller are discussed in Section 4. In Section 6, the remarks concluded from this study are described.

## 2. DOF Human Musculoskeletal Arm Model

**Figure 1:**
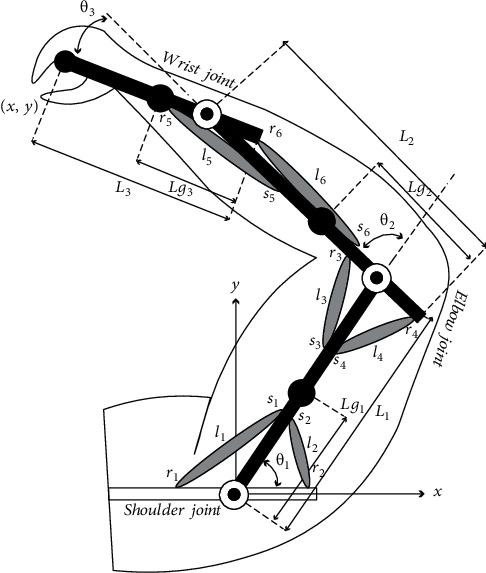
Schematic view of the 3-DOF musculoskeletal model for the hand.

The 3-DOF human musculoskeletal arm model used in this study consisting of three solid links and six monoarticular muscles is shown in Figure 1. Since this arm moves on the horizontal plane, the effect of the gravity force can be ignored. As shown in Figure 1, this model consists of six muscles that can only apply tensile forces so that each joint moves by some of these related muscles. Muscles are assumed to be without weight and designed based on the Hill model, which are directly connected to links as

(1) $$\begin{array}{c} f_{\text{m}}=P\bar{\alpha }-P\left\{A\left(\bar{\alpha }\right)C+C_{0}\right\}\dot{l},\\ \left[\begin{array}{c} \bar{\alpha }=\left({\bar{\alpha }}_{1},{\bar{\alpha }}_{2},\cdots ,{\bar{\alpha }}_{6}\right)\;\epsilon \;R^{6},\\ P=\mathrm{diag}\left(p_{1},p_{2},\cdots ,p_{6}\right)\;\epsilon \;R^{6\times 6},\\ A\left(\bar{\alpha }\right)=\mathrm{diag}\left({\bar{\alpha }}_{1},{\bar{\alpha }}_{2},\cdots ,{\bar{\alpha }}_{6}\right)\;\epsilon \;R^{6\times 6},\\ C=\mathrm{diag}\left(c_{1},c_{2},\cdots ,c_{6}\right)\;\epsilon \;R^{6\times 6},\\ C_{0}=\mathrm{diag}\left(c_{01},c_{02},\cdots ,c_{06}\right)\;\epsilon \;R^{6\times 6}. \end{array}\right. \end{array}$$

From Ref. [13], parameter *f*_m_ is the muscles' contractile force, which is the nonlinear function of the muscle's contractile velocity and the control input produced in the central nervous system.

Considering *L*_1_, *L*_2_ , and *L*_3_ to be the first, second, and third links, respectively, as well as their relation angle with respect to the *x*-axis, first link, and second link as *θ*_1_, *θ*_2_, and *θ*_3_, respectively, the arm's end effector position to the joint angles is expressed by the following equation:

(2) $$\begin{array}{c} X=J\dot{\left(\theta \right)=\left[\begin{array}{c} L_{1}\cos\left(\theta_{1}\right)+L_{2}\cos\left(\theta_{1}+\theta_{2}\right)+L_{3}\cos\left(\theta_{1}+\theta_{2}+\theta_{3}\right)\\ L_{1}\sin\left(\theta_{1}\right)+L_{2}\sin\left(\theta_{1}+\theta_{2}\right)+L_{3}\sin\left(\theta_{1}+\theta_{2}+\theta_{3}\right) \end{array}\right]}\in R^{2}. \end{array}$$

Defining length vectors of the muscles as *l* = [*l*_1_ *l*_2_ *l*_3_ *l*_4_ *l*_5_ *l*_6_]^*T*^ results in

(3) $$\begin{array}{c} l=W\left(\theta \right)=\left[\begin{array}{c} r_{1}\left(\pi -\theta_{1}-a\cos\left(\frac{r_{1}}{s_{1}}\right)\right)+\sqrt{s_{1}^{2}-r_{1}^{2}}\\ r_{2}\left(\frac{\pi }{2}+\theta_{1}-a\cos\left(\frac{r_{2}}{s_{2}}\right)\right)+\sqrt{s_{2}^{2}-r_{2}^{2}}\\ r_{3}\left(\pi -\theta_{2}-a\cos\left(\frac{r_{3}}{s_{3}}\right)\right)+\sqrt{s_{3}^{2}-r_{3}^{2}}\\ r_{4}\left(\frac{\pi }{2}+\theta_{2}-a\cos\left(\frac{r_{4}}{s_{4}}\right)\right)+\sqrt{s_{4}^{2}-r_{4}^{2}}\\ r_{5}\left(\frac{\pi }{2}+\theta_{3}-a\cos\left(\frac{r_{5}}{s_{5}}\right)\right)+\sqrt{s_{5}^{2}-r_{5}^{2}}\\ r_{6}\left(\frac{\pi }{2}+\theta_{3}-a\cos\left(\frac{r_{6}}{s_{6}}\right)\right)+\sqrt{s_{6}^{2}-r_{6}^{2}} \end{array}\right]\in R^{6}, \end{array}$$

where *r*_1−6_ and *s*_1−6_ represent the torque levers, as shown in Figure 1. The following equations are obtained by taking the time derivative of equations (2) and (3) with respect to time:

(4) $$\begin{array}{c} \dot{X}=J\dot{\theta }, \end{array}$$

(5) $$\begin{array}{c} \dot{l}=W^{T}\dot{\theta }. \end{array}$$

$\dot{X}={\left[\begin{array}{cc} \dot{x} & \dot{y} \end{array}\right]}^{T}$ is the end effector velocity of the arm, $\dot{\theta }={\left[\begin{array}{ccc} \dot{\theta } & {\dot{\theta }}_{2} & {\dot{\theta }}_{3} \end{array}\right]}^{T}$ is the angular velocity of joints, and $\dot{l}={\left[\begin{array}{cccccc} {\dot{l}}_{1} & {\dot{l}}_{2} & {\dot{l}}_{3} & {\dot{l}}_{4} & {\dot{l}}_{5} & {\dot{l}}_{6} \end{array}\right]}^{T}$ represents the stretch rate of muscles. Also, *J* ∈ *R*^2×3^ is the Jacobian matrix that shows the relation between the linear velocities of the arm's end effector and angular velocities while *W*^*T*^ ∈ *R*^6×3^ is the Jacobian matrix, which relates the contractile rate of muscles to the angular velocity of the joints as

(6) $$\begin{array}{c} J=\left[\begin{array}{ccc} J_{11} & J_{12} & J_{13}\\ J_{21} & J_{22} & J_{23} \end{array}\right],\\ J_{11}=-L_{1}\sin\left(\theta_{1}\right)-L_{2}\sin\left(\theta_{1}+\theta_{2}\right)-L_{3}\sin\left(\theta_{1}+\theta_{2}+\theta_{3}\right),\\ J_{12}=-L_{2}\sin\left(\theta_{1}+\theta_{2}\right)-L_{3}\sin\left(\theta_{1}+\theta_{2}+\theta_{3}\right),\\ J_{13}=-L_{2}\sin\left(\theta_{1}+\theta_{2}\right)-L_{3}\sin\left(\theta_{1}+\theta_{2}+\theta_{3}\right),\\ J_{21}=L_{1}\cos\left(\theta_{1}\right)+L_{2}\cos\left(\theta_{1}+\theta_{2}\right)+L_{3}\cos\left(\theta_{1}+\theta_{2}+\theta_{3}\right),\\ J_{22}=L_{2}\cos\left(\theta_{1}+\theta_{2}\right)+L_{3}\cos\left(\theta_{1}+\theta_{2}+\theta_{3}\right),\\ J_{23}=L_{3}\cos\left(\theta_{1}+\theta_{2}+\theta_{3}\right),\\ W=\left[\begin{array}{cccccc} -r_{1} & r_{2} & 0 & 0 & 0 & 0\\ 0 & 0 & -r_{3} & r_{4} & 0 & 0\\ 0 & 0 & 0 & 0 & -r_{5} & r_{6} \end{array}\right]. \end{array}$$

By assuming *J* as a full-rank matrix, the inverse of equations (2) and (4) is obtained as follows:

(7) $$\begin{array}{c} \theta =G_{x}^{-1}\left(x\right)\in R^{2}, \end{array}$$

(8) $$\begin{array}{c} \dot{\theta }=J^{-1}\dot{x}\in R^{2}. \end{array}$$

*G*_*x*_^−1^(*x*) represents a vector with nonlinear functions, which shows the inverse kinematics from the task space to joint space. Also, *J*^−1^ shows the inverse kinematics from the task space velocity to the joint's angular velocity. By substituting equation (8) into equations (3) and (5), we can state that

(9) $$\begin{array}{c} l=G_{\iota }\left(G_{x}^{-1}\left(x\right)\right)\in R^{6}, \end{array}$$

(10) $$\begin{array}{c} \dot{l}=W^{T}J^{-1}\dot{x}\in R^{6}. \end{array}$$

Equation (9) demonstrates the inverse kinematics from the task space to the muscle space, which is applied to the controller's feedforward behavior.

By applying the principle of virtual work, the work done by muscle torque is defined as follows:

(11) $$\begin{array}{c} T=Wf_{\text{m}}\in R^{2}, \end{array}$$

where $f_{\text{m}}={\left[\begin{array}{cccccc} f_{1} & f_{2} & f_{3} & f_{4} & f_{5} & f_{6} \end{array}\right]}^{T}$ is the vector representing the tensile forces of muscles and $T={\left[\begin{array}{ccc} T_{1} & T_{2} & T_{3} \end{array}\right]}^{T}$ is the joint torque vector.

By assuming that *W* ∈ *R*^3×6^ is a row full-rank matrix during movement, the inverse of equation (11) is expressed as follows:

(12) $$\begin{array}{c} f_{\text{m}}=W^{+}T\in R^{6}, \end{array}$$

(13) $$\begin{array}{c} W^{+}=W^{T}{\left(WW^{T}\right)}^{-1}\in R^{6\times 3}. \end{array}$$

Besides, the static relation between *T* and the output vector of forces applied to the arm's endpoint in the space *F* ∈ *R*^2^ is expressed as follows:

(14) $$\begin{array}{c} T=J^{T}F\in R^{3}. \end{array}$$

By substituting equation (14) into equation (13), it is concluded that

(15) $$\begin{array}{c} f_{\text{m}}=W^{+}J^{T}F\in . \end{array}$$

Equation (15) demonstrates the static inverse relation between *f*_m_ and *F*.

## 3. Iterative Learning Control

An ILC strategy of the PI type has been introduced in Reference [14] to trace an arbitrary time-dependent trajectory using the robotic arm model. The errors related to the position and velocity in a test are stored to be tuned for the next test by an input correction strategy. The data stored in the first step are multiplied by a factor and added to the input in the next test. Implementing a simple task space feedback control for a 2-DOF arm is considered by Tahara et al. to address the muscle space redundancy problem on the contractile output force [15]. They also studied multiple space variables to enhance the robustness of the 2-DOF arm exposed to sensory noises. Despite the nonlinear equations of the human arm's motion, the suggested method sufficiently improves the system's robustness regarding the traditional ILC methods [16]. Therefore, the proposed method is considered in our study. As discussed in the previous section, to compensate for the iterative learning controller's input, there are three representatives of the state space, namely, muscle space, joint space, and task space. Therefore, any space that can better compensate for the control input is of great importance in achieving the desired performance. Furthermore, it should be noted that many noises cause damage to sensory information, and its huge impact on the movement of the musculoskeletal system is inevitable. Therefore, the system's robustness to deal with the noise varies depending on the space in which the system is modeled. A new control strategy based on iterative learning, which uses the sum of state-space variables, is employed to improve the robustness of the system against noise. In the present paper, a case study is performed by considering the task space and muscle space as the spaces for feedback and feedforward behaviors, respectively. The control input of the muscles in the *i*th test is defined as follows:

(16) $$\begin{array}{c} u_{i}=-{W_{i}}^{+}{J_{i}}^{T}\left(K_{p}\Delta x_{i}-K_{\upsilon }\Delta {\dot{x}}_{\text{i}}\right)+\upsilon_{i}, \end{array}$$

where index *i* represents the test number, *K*_*p*_ = diag[*k*_*p*1,_*k*_*p*2_] ∈ *R*^2×2^ > 0 and *K*_*υ*_ = diag[*k*_*υ*1,_*k*_*υ*2_] ∈ *R*^2×2^ > 0 are the feedback coefficients of position and velocity in the task space, and *υ*_*i*_ is the feedforward parameter obtained from the iterative learning process. The error of position and velocity is defined as Δ*x*_*i*_ = *x*_*i*_ − *x*_*d*_ ∈ *R*^2^ and $\Delta {\dot{x}}_{i}={\dot{x}}_{i}-{\dot{x}}_{d}\in R^{2}$, respectively; *x*_*d*_ and ${\dot{x}}_{d}$ also represent the end effector's position and velocity, respectively. The feedforward parameter, *υ*_*i*_ ∈ *R*^6^, is not designed in the task space similar to feedback behavior, but it is modeled in the muscle space and updated as follows:

(17) $$\begin{array}{c} \upsilon_{i}=\left\{\begin{array}{cc} 0, & \begin{array}{cc} \; & i=1, \end{array}\\ \left(1-\beta \right)\upsilon_{i-1}-\left(\Phi \Delta \iota_{i-1}+\Psi \Delta {\dot{\iota }}_{i-1}\right), & \begin{array}{cc} \; & i>1 \end{array}, \end{array}\right. \end{array}$$

where *Φ* = diag[*ϕ*_1_, *ϕ*_2_, ⋯, *ϕ*_6_] ∈ *R*^6×6^ > 0 and Ψ = diag[*ψ*_1_, *ψ*_2_, ⋯, *ψ*_6_] ∈ *R*^6×6^ > 0 are the coefficient matrices of position and velocity, respectively; besides, the position error is defined as Δ*ι*_*i*_ = *ι*_*i*_ − *ι*_*d*_ and the velocity errors in the muscle space are expressed as $\Delta {\dot{\iota }}_{i}={\dot{\iota }}_{i}-{\dot{\iota }}_{d}$. *ι*_*d*_ ∈ *R*^6^ and ${\dot{\iota }}_{d}\in R^{6}$ are the length of muscles and their contraction rate relative to the position and velocity of the end effector, respectively. These parameters are obtained by calculating the inverse dynamic as

(18) $$\begin{array}{c} \Delta \iota_{i}=G_{\iota }\left(G_{x}^{-1}\left(x_{i}\right)\right)-G_{\iota }\left(G_{x}^{-1}\left(x_{d}\right)\right),\\ \Delta {\dot{\iota }}_{i}=W_{i}^{T}J_{i}^{-1}\Delta {\dot{x}}_{i}. \end{array}$$

In this study, the Gaussian noise is used as a noise which is applied to sensory information. An error in the initial conditions of two consecutive tests and dynamic oscillations due to different types of noises causes the general system to be unstable using the iterative learning controller. Therefore, to overcome these noises, Suetani and Morimoto [11] introduced a forgetting factor to update the iterative learning controller. Using this forgetting factor ensures that the final converged trajectory after good learning is in the desired trajectory neighborhood. In equation (17), *β* is the forgetting factor that has to satisfy the condition of 0 < *β* < 1. It is assumed that the muscle's length and end effector position and velocity signals include Gaussian noise individually. Due to Refs. [15, 17], the magnitude of the noise existing in the end effector's position and velocity is 4% of real data, and the magnitude of noise existing in the length of the muscle and its contraction rate is 50% of the real data. This is because the data related to the end effector's position and velocity are obtained through observation, which is relatively accurate. However, the data related to the muscle's length and contraction rate are received through the muscular bulk which has large electrical noise leading to inaccurate results [18].

## 4. Results

The simulation results are presented in this section. Tables 1 and 2 demonstrate the numerical values associated with the 3-link model and the values related to the muscles' physical properties, respectively. Also, the coefficients of the controller are listed in Table 3.

The controller is aimed at tracing a semicircular trajectory. Therefore, we consider the following trajectory:

(19) $$\begin{array}{c} \left\{\begin{array}{c} x=0.2+0.1\cos\left(t\right),\\ y=0.55+0.1\sin\left(t\right). \end{array}\right. \end{array}$$

The simulation's total time is assumed to be *T* = *πs*, and the hand is initially located at point (−0.1,0.55). Therefore, during the aforementioned period, the robot is expected to cover the semi-semicircular trajectory fully. For evaluating the robustness of the presented model against uncertainties, the simulation parameters have been changed by 5%. To compare the controller's performance with similar counterparts, the model control results are compared to the neuro-fuzzy control method presented in our other paper [19]. In the cited article, the similar given trajectory was precisely followed by the muscle optimization, and the results concluded appropriate compliances with the hand's natural motion. The model simulation was performed in MATLAB version 2021a running on an Intel Core i7 (2.8 GHz and 16 Gb RAM). For all simulations, the variable-step MATLAB ODEs solver ode45 with relative solver tolerance 1 × 10^−4^ was implemented, which took 4.817 s for the ILC controller compared to 10.045 s for the neuro-fuzzy controller.

**Figure 2:**
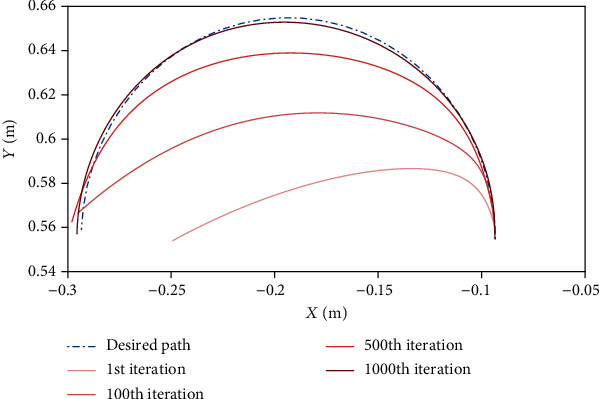
The trajectories passed by the model per 1000 iterations.

**Figure 3:**
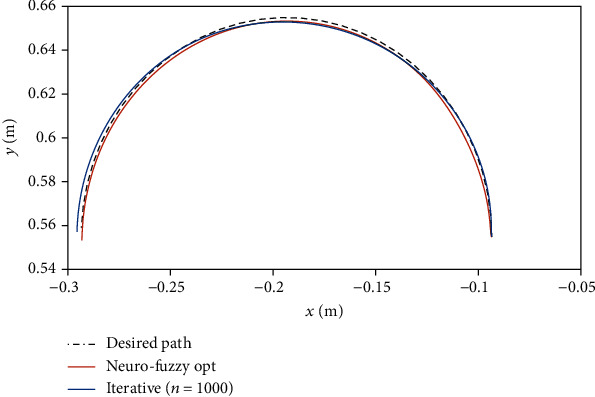
Comparison of trajectories covered by the neuro-fuzzy adaptive controller and ILC.

Figure 2 depicts different trajectories that the controller has taken over 1000 iterations to reach the desired trajectory. As can be seen, as the controller's performance is improved, the error is reduced gradually. Therefore, in iteration no. 1000, the trajectory is adjusted to the desired trajectory. Such a process is similar to learning and muscle memory that can perfectly go through a trajectory with practice and repetition. The trajectories that both controllers have gone through at the same time are shown in Figure 3. The results show that the proposed controller has better performance. In other words, if we exceed 1000 iterations in training the controller, we will observe a further improvement in the controller results. However, it should be noted that the simulation time increases with increasing the number of iterations.

**Figure 4:**
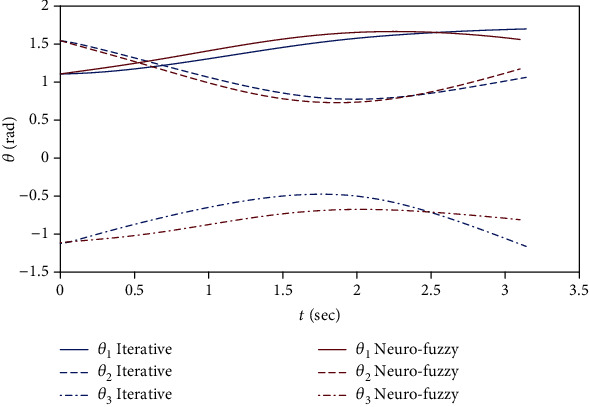
Comparison of joint displacement between the neuro-fuzzy adaptive controller and ILC.

Figure 4 displays the displacement of different joints during the movement scenario. The displacement of joints is similar to another. The adaptive controller performance is based on the optimization of the cost function and the iterative controller performance on learning; hence, Figure 4 designates that the proposed controller performance is acceptable compared to the adaptive controller's performance using the neuro-fuzzy adaptive controller.

**Figure 5:**
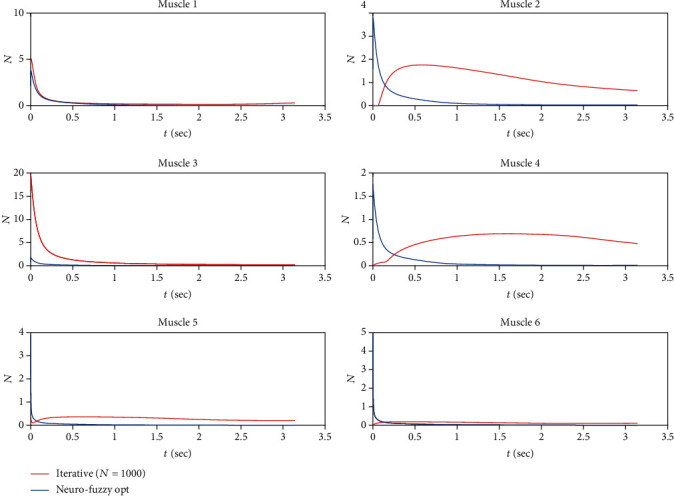
Comparison of the generated forces using the neuro-fuzzy adaptive controller and ILC.

Finally, the magnitude of forces applied to each muscle during the desired trajectory is illustrated in Figure 5. The neuro-fuzzy controller uses muscle optimization; therefore, its force diagrams are much more ideal. On the other hand, the ILC controller has a more smooth bell-shaped profile similar to the agonist-antagonist paired muscles involved in the natural movements of the human body. However, both controllers have similar patterns. In addition, muscle forces in both methods are in the adequate range for the human body, where the ILC method has almost fewer force values.

## 5. Discussion

The controller has two pieces, the first of which is feedback input comprising task space variables, while the other part is the feedforward input, which is made up of muscle space parameters gained through the iterative learning algorithm. Although all controller gains are tuned in all iterations, the simulation results demonstrated that the hand endpoint in the first iteration is significantly different from the 1000^th^ iteration. Despite the nonlinear equations of muscles, the controller could pass through the desired trajectory after the 1000^th^ iteration. In addition, the path tracking error has considerably been mitigated by increasing the repetition number. The use of different variable spaces in conjunction with the learning algorithm was the primary reason for the sufficient accuracy of path tracking in the proposed controller.

Furthermore, the travel time of the simulation was reduced to half using the ILC controller, compared to the neuro-fuzzy one following the 1000th repetition of the desired path. Force values for the given path were also in the sufficient force ranges of the human hand muscles. In most muscles, the endpoint passed through the given trajectory with a much lower force than the neuro-fuzzy controller. These results characterize the efficiency of this controller for musculoskeletal modeling in the human body. As a future study, we intend to conduct movement trials in actual and uncontrolled conditions using EMG signals plus effective technologies such as user-friendly contactless path recognition to increase the method's productivity [5].

## 6. Conclusion

The controller's performance was improved by iterating learning, and subsequently, the related error was reduced so that the final trajectory that has gone through simulation is practically adjusted to the desired trajectory. Such a process is similar to learning and muscle memory that can lead to perfectly going through a trajectory with practice and repetition. The quantitative comparison between the iterative learning controller and neuro-fuzzy controller results suggested that the proposed controller has a better performance. In other words, if we exceed 1000 iterations in training the controller, we will observe a further improvement in the controller results. However, it should be noted that the time required for solving the problem increases by increasing the number of iterations. By comparing the forces generated in the muscles for both controllers, it was observed that the maximum value of these forces for the current controller was less than that of the adaptive controller, although the average of generated force is higher for the current controller. Considering that the muscle forces' optimization is one of the design indicators in adaptive controllers, it was not considered in the proposed method. Here, it was important that the controller can successfully guide the model on the desired trajectory in the presence of system uncertainties, and the forces applied to the muscles are in the desired range.

## Figures and Tables

**Table 1:** Numerical parameters of the model.

	Length (m)	Mass (kg)	Inertial moment (kg*·*m^2^)	CoM position (m)
1^st^ link	0.31	1.93	0.0141	0.165
2^nd^ link	0.27	1.32	0.0120	0.135
3^nd^ link	0.15	0.35	0.0010	0.075

**Table 2:** Geometric parameters of the muscles.

Muscle	Value (m)	
*l*_1_	*r*_1_ = 0.055	*s*_1_ = 0.080
*l*_2_	*r*_2_ = 0.055	*s*_2_ = 0.080
*l*_3_	*r*_3_ = 0.030	*s*_3_ = 0.120
*l*_4_	*r*_4_ = 0.030	*s*_4_ = 0.120
*l*_5_	*r*_5_ = 0.035	*s*_5_ = 0.220
*l*_6_	*r*_6_ = 0.040	*s*_6_ = 0.250

**Table 3:** The parameters associated with the controller.

Parameter	Value
Feedback gain	*K*_*p*_ = [8080]
Feedback gain	*K*_*v*_ = [5050]
Learning gain	*Φ*_1_ = ⋯ = *Φ*_6_ = 250
Learning gain	Ψ_1_ = ⋯ = Ψ_6_ = 140
Forgetting factor	*β* = 0.3

## Data Availability

The data is extracted from our other paper entitled “The Optimal Adaptive-Based Neuro-Fuzzy Control of the 3-DOF Musculoskeletal System of Human Arm in A 2D Plane.”
